# 1938. CoviLiv™, a Novel Intranasal Live-Attenuated COVID-19 Vaccine Candidate, Induces Robust Humoral and Cellular Immunity in First-In-Human Clinical Trial CDX-CoV-001

**DOI:** 10.1093/ofid/ofad500.2469

**Published:** 2023-11-27

**Authors:** Johanna K Kaufmann, Keri Wyllie, Yiwen Zhao, Lasmy Tea, Sybil Tasker, Leena R Yeolekar, Rajeev Dhere, Steffen Mueller

**Affiliations:** Codagenix Inc., Cambridge, MA; Codagenix Inc., Cambridge, MA; Codagenix Inc., Cambridge, MA; Codagenix Inc., Cambridge, MA; Codagenix Inc., Cambridge, MA; Serum Institute of India Pvt. Ltd., Pune, Maharashtra, India; Serum Institute of India Pvt. Ltd., Pune, Maharashtra, India; Codagenix, Inc, Farmingdale, NY

## Abstract

**Background:**

CoviLiv is a novel intranasal live-attenuated COVID-19 vaccine candidate, derived from SARS-CoV-2/Wuhan that was synthetically engineered utilizing Codagenix’ codon pair deoptimization platform. CDX-CoV-001 was a randomized, double-blind, placebo-controlled dose-escalation study in healthy adults, that established its safety and tolerability (NCT04619628). Here, we characterize the immune response to CoviLiv in participants in the high-dose cohort of 5x10^6^ pfu (n=6/group).

**Methods:**

Humoral immune responses were characterized in sera (days 1, 29 and 57) with a spike-specific IgG ELISA and both microneutralization (MNT) and pseudovirus neutralization (PVN) assays. T cell functionality was analyzed in PBMCs (days 1 and 36) using interferon-γ (IFNγ) ELISpot and intracellular cytokine staining (ICS) after restimulation with peptide pools for SNMO or spike, as well as T cell receptor (TCR) sequencing combined with *in silico* mapping to TCRs with known antigen specificity.

**Results:**

After 2 doses of CoviLiv, all participants exceeded a 2-fold increase in spike-specific IgG with a geometric mean fold rise of 19.5 (95% CI 3.4-113.8) on day 57. Neutralizing antibodies at this timepoint were induced 2.6-fold (CI 1.0-7.0) and 4.9-fold (CI 1.4-16.6) using MNT and PVN. On day 36 post-vaccination, IFNγ response by ELISpot after restimulation with the SNMO peptide pool increased 4.5-fold (CI 2.8-7.4) in the 2-dose cohort and 2.5-fold (CI 1.4-4.2) in the 1-dose cohort. No increase in IFNγ response was observed after placebo vaccination or in any group after restimulation with spike-only peptides. ICS confirmed significant regimen-dependent SNMO-specific increases in IFNγ, IL-2 and TNFα response especially in the CD4^+^ T cell subset, including induction of polyfunctional CD4^+^ T cells. TCR repertoire mapping revealed increases in both depth and breadth of CD4^+^ T cell clones specific for spike, nucleocapsid phosphoprotein and membrane glycoprotein. CD8^+^ T cell responses were less pronounced with CoviLiv-induced responses directed against nucleocapsid and ORF1ab.

**Conclusion:**

Two doses of CoviLiv induced a poly-antigenic immune response with specificity to targets beyond spike, including highly conserved viral proteins, highlighting the value of this differentiated vaccine candidate.

**Disclosures:**

**Johanna K. Kaufmann, PhD**, Codagenix Inc.: Salaried employee|Codagenix Inc.: Ownership Interest **Keri Wyllie, MPA**, Codagenix Inc.: Salaried employee|Codagenix Inc.: Ownership Interest **Yiwen Zhao, PhD**, Codagenix Inc.: Salaried employee **Lasmy Tea, MS, MPH**, Codagenix Inc.: Salaried employee|Codagenix Inc.: Ownership Interest **Sybil Tasker, MD, MPH, FIDSA**, Codagenix Inc.: Advisor/Consultant|Codagenix Inc.: Salaried employee|Codagenix Inc.: Ownership Interest|Inventprise: Salaried employee|Inventprise: Ownership Interest **Leena R. Yeolekar, PhD**, Serum Institute of India Pvt. Ltd.: Salaried employee **Rajeev Dhere, PhD**, Serum Institute of India Pvt. Ltd.: Salaried employee **Steffen Mueller, PhD**, Codagenix Inc.: Board Member|Codagenix Inc.: Patent owner|Codagenix Inc.: Salaried employee|Codagenix Inc.: Ownership Interest

